# Personal

**Published:** 1899-09

**Authors:** 


### PERSONAL.

DR. L. S. MCMURTRY, of Louisville, president of the faculty of the Hospital College of Medicine, medical department of Central University of Kentucky, will deliver the address upon surgery at the meeting of the Mississippi Valley Medical Association, to be held at Chicago, October 3-6, 1899. Dr. H. H. Mudd, of St. Louis, was elected to this office, but owing to his absence in Europe, will be unable to perform the duty. Therefore the president, Dr. Duncan Eve, of Nashville, has appointed Dr. McMurtry in his stead. It is needless to add that an interesting and instructive address from Dr. McMurtry may be expected.

DR. C. G. STIVERS, formerly of Niagara Falls, now of Los Angeles, Cal., has become assistant editor and business manager of the Southern California *Practitioner* and will have a general oversight, both editorially and financially. The *Practitioner* in its issue for July says : "Dr. Stivers is a graduate of the University of Pennsylvania, after which he spent eighteen months as an interne in the well-known Blockley Hospital, Philadelphia. Since his leaving the hospital he has practised medicine in Niagara Falls until about a year ago, when he located in Los Angeles. Since coming to this city he has gained the esteem of the physicians of Los Angeles, and his association with the *Practitioner* is a matter of congratulation for the profession of Southern California."

---

Dr. Herman Mynter, of Buffalo, sailed on the Friederich der Grosse, August 10, 1899, for a visit to Denmark and Norway. At Copenhagen he will read a paper before the Royal Medical Society on the Etiology and pathology of appendicitis and their bearing upon its treatment. Denmark, it is well known, still maintains that appendicitis is a medical disease and that the strict opium treatment is the correct one and Dr. Mynter has been challenged to prove the opposite. We have little doubt but that he will be able to convince his Denmark confrères of their error. He will return during the first week of October to resume his professional work.

---

Dr. A. Martin, the well-known gynecologist of Berlin, and whose fame in that department of medicine is world-wide, has been appointed ordinary professor of gynecology in the University of Greifswald. This is in accordance with the established German method of advancing subordinate teachers to first place as vacancies occur.

---

Dr. Henry Jones Mulford, of Buffalo, announces the removal of his office to "The Colonial," 401 Delaware avenue. Hours: 10 a. m. to 12 m., 2 to 4 p. m.; Sunday, 12 m. to 1 p. m. Residence, The "Elmwood Heights," 597 Elmwood Avenue. Practice limited to diseases of nose, throat and ear.

---

Dr. and Mrs. William G. Krauss, who have been summering in Europe, have returned to Buffalo after a most delightful tour. Dr. Krauss barely reached Buffalo in time to hasten to Columbus to preside at the meeting of the American Microscopical Society.

---

Dr. Floyd S. Crego, of Buffalo, who has been spending some months in the clinics of Europe, has returned and taken temporary quarters at the Trubee, 414 Delaware avenue. Early in September he will reoccupy his residence, 469 Delaware avenue, corner of Virginia street.

---

Dr. F. D. Thompson, of Forth Worth, Texas, recently paid a short visit to Buffalo en route to the coast of Maine to visit his son who is at school in that state. Dr. Thompson, who is one of the most prominent physicians in Texas, pays frequent visits to the North, this being the second one since January 1, 1899.

---

Dr. Max Breuer, of Buffalo, a former surgeon in the Imperial Army of Germany, has just been decorated with the Cross of the Legion of Honor for saving the life of a French seaman. He was on board a German vessel, and hearing that a French seaman was on board a British ship suffering from a wound in the arm, he at once went to the man's assistance. He amputated the arm, and so saved the sailor's life. This prompt action came to the notice of the French Minister for Foreign Affairs, with the result that the German doctor has been decorated. Dr. Breuer is now on a visit to his native country, but will return and resume his practice early in the autumn.

---

Dr. Max Sanger, the well-known gynecologist of Leipzig, has accepted the chair of obstetrics and gynecology at the German university of Prague in succession to Professor Rosthorn, who was recently transferred to Innsbrück. —*Med. Bulletin.*

---

Dr. Charles A. Wall, of Buffalo, who has been touring with his family in Europe the past summer, has returned and resumed his practice.

